# Cube attacks on round-reduced TinyJAMBU

**DOI:** 10.1038/s41598-022-09004-3

**Published:** 2022-03-29

**Authors:** Wil Liam Teng, Iftekhar Salam, Wei-Chuen Yau, Josef Pieprzyk, Raphaël C.-W. Phan

**Affiliations:** 1 School of Computing and Data Science, Xiamen University Malaysia, Sepang, 43900 Malaysia; 2 Data61, Commonwealth Scientific and Industrial Research Organisation, Marsfield, NSW 2122 Australia; 3 Institute of Computer Science, Polish Academy of Sciences, 01-248 Warsaw, Poland; 4 School of IT, Monash University, Subang Jaya, 47500 Malaysia; 5 Department of Software Systems & Cybersecurity, Faculty of IT, Monash University, Melbourne, VIC 3800 Australia

**Keywords:** Computational science, Computer science

## Abstract

Lightweight cryptography has recently gained importance as the number of Internet of things (IoT) devices connected to Internet grows. Its main goal is to provide cryptographic algorithms that can be run efficiently in resource-limited environments such as IoT. To meet the challenge, the National Institute of Standards and Technology (NIST) announced the Lightweight Cryptography (LWC) project. One of the finalists of the project is the TinyJAMBU cipher. This work evaluates the security of the cipher. The tool used for the evaluation is the cube attack. We present five distinguishing attacks DA1–DA5 and two key recovery attacks KRA1–KRA2. The first two distinguishing attacks (DA1 and DA2) are launched against the initialisation phase of the cipher. The best result achieved for the attacks is a distinguisher for an 18-bit cube, where the cipher variant consists of the full initialisation phase together with 438 rounds of the encryption phase. The key recovery attacks (KRA1 and KRA2) are also launched against the initialisation phase of the cipher. The best key recovery attack can be applied for a cipher variant that consists of the full initialisation phase together with 428 rounds of the encryption phase. The attacks DA3–DA5 present a collection of distinguishers up to 437 encryption rounds, whose 32-bit cubes are chosen from the plaintext, nonce, or associated data bits. The results are confirmed experimentally. A conclusion from the work is that TinyJAMBU has a better security margin against cube attacks than claimed by the designers.

## Introduction

In recent years there has been an upwards trend for the usage of Internet-of-Things (IoT) devices, especially in the healthcare and manufacturing industries. The trend has led to IoT devices being virtually omnipresent and more interconnected than ever before. Consequently, there is a need to tighten the security of IoT devices. Unfortunately, traditional cryptographic algorithms are designed for resource-rich environments. In contrast, IoT devices are usually lightweight and they operate in resource-constrained environments. Thus using traditional cryptographic algorithms for IoT devices causes a significant performance degradation^1^. To mitigate the mismatch, a new branch of cryptography has emerged in recent years. It is called lightweight cryptography (LWC). Its main goal is to design cryptographic algorithms that can be run efficiently in IoT (resource-constrained) environments.

The LWC Standardisation Project^2^ is an initiative of the US National Institute of Standards and Technology (NIST). It was launched in 2013 and aims to evaluate and select standards for LWC. The project is currently in its final round^3^. Ten finalists were announced in March 2021. They are: ASCON, Elephant, GIFT-COFB, Grain-128AEAD, ISAP, PHOTON-Beetle, Romulus, Sparkle, TinyJAMBU and Xoodyak. There is a need for a third-party analysis of the LWC finalists. The analysis provides a crucial service to the community at large as it helps to determine secure and efficient LWC standards. This work contributes to the analysis and evaluates security of the TinyJAMBU cipher. In particular, it assesses the strength of TinyJAMBU against cube attacks.

TinyJAMBU^4^ is a sponge-based stream cipher that provides authenticated encryption with associated data (AEAD). There are two versions of the cipher. The first is the original submission to the LWC Project. The second was released in May 2021 and is called TinyJAMBUv2^5^. The cube attacks presented in the paper are applied against the first version of TinyJAMBU. However, some attacks (DA2 with reduced cube space, KRA2, DA3, DA4, and DA5) are still applicable to TinyJAMBUv2 as the tweaks in the second version do not affect our attacks. For the rest of the paper, unless explicitly specified, TinyJAMBU refers to the first version of the cipher.

### Cube attacks against AEAD stream ciphers

The cube attack is a generalisation of the higher-order differential attack^6^ and the algebraic IV differential attack (AIDA)^7^. It was proposed by Dinur and Shamir at EUROCRYPT 2009^8^. The attack sums output values of a black box polynomial ${\mathcal {P}}$ over all possible values of a chosen collection of input variables. It aims to reduce the degree of ${\mathcal {P}}$. The collection of input variables is called a cube ${\mathcal {C}}$. The cube is uniquely determined by a set *I* of input variable indices. A polynomial ${\mathcal {P}}_{S(I)}$ obtained after summation over ${\mathcal {C}}$ is called a superpoly. In 2009 Dinur and Shamir applied the cube attack against the Trivium stream cipher^8^. Since then, the attack has been used to analyse many other stream ciphers, see references^9–18^, for example.

TinyJAMBU is a sponge-based AEAD stream cipher. When considering an AEAD stream cipher, the cube attack may be applicable to different cipher phases. A typical stream cipher has the following phases: initialisation, associated data processing, encryption, finalisation, decryption and verification. Application of cube attacks against different cipher phases requires specific security assumptions. In general, each attack aims to recover some secret information about the cipher. The following list identifies typical attacks against AEAD stream ciphers.

- Key recovery attacks (KRA)—they aim to retrieve the superpolies of cubes, which include variables of a secret key. The attack is typically applied against the initialisation phase, where the key is input into the internal states with some public variables. In the case of TinyJAMBU, key recovery cube attacks can be launched against any phase. This is due to the fact that the key bits are input to the internal state of the cipher during all phases.
- State recovery attacks (SRA)—they target superpolies that include internal state variables. They are applicable when the superpoly depends on both few internal states and some public variables at a particular time instance (clock).
- Distinguishing attacks (DA)—they allow to differentiate a stream cipher from a truly random one. They work if there is a superpoly, which becomes a constant (zero or one) after summing over all cube values. Such cubes are also called cube testers^9^.
- Known plaintext attacks (KPA)—it is assumed that an adversary is able to read plaintexts and associated data but is not able to change them. Consequently, cubes chosen by the adversary can include neither plaintext nor associated data bits. They, however, can include initialisation vector/nonce bits. In this case, we deal with a chosen initialisation vector attack. For TinyJAMBU, its nonces contain 96 bits. Thus, an adversary may select cubes from the nonce bits.
- Chosen plaintext attacks (CPA)—it is assumed that an adversary can not only read plaintext and associated data but also it is able to modify them at will. This means that the adversary can choose cubes that include both plaintext and associated data bits (apart from initialisation vector bits).

### Our contributions

Note that attacks presented in the work are against round-reduced versions of TinyJAMBU. We apply five distinct strategies for cube selection that allow us to construct appropriate distinguishers. Our five distinguishing attacks (DA1–DA5) can be launched against both initialisation and encryption phases of the cipher. DA1 and DA2 are applied against the cipher initialisation phase. The other attacks (DA3–DA5) are implemented against the encryption phase. Table 1 shows a summary of our results.

**Table 1:** Summary of our results. The variables $v_{i}, m_{i}, d_{i}$ are referring to the nonce bits, plaintext bits, and associated data bits, respectively.

Attack	Selection of cube variables	Cube size	No. of rounds (reduced)
DA1 & KRA1	${\mathcal {C}} \in \{ v_{0}, \cdots , v_{63} \}$	3	2176 initialisation rounds & 0 encryption rounds
DA2 (full cube space)	${\mathcal {C}} \in \{ v_{0}, \cdots , v_{95} \}$	25	2176 initialisation rounds & 416 encryption rounds
DA2 (reduced cube space)	${\mathcal {C}} \in \{ v_{64}, \cdots , v_{95} \}$	18	2176 initialisation rounds & 438 encryption rounds
KRA2	${\mathcal {C}} \in \{ v_{64}, \cdots , v_{95} \}$	14	2176 initialisation rounds & 428 encryption rounds
DA3	${\mathcal {C}} \in \{ m_{0}, \cdots , m_{31} \}$	32	437 encryption rounds
DA4	${\mathcal {C}} \in \{ v_{64}, \cdots , v_{95} \}$	32	437 encryption rounds
DA5	${\mathcal {C}} \in \{ d_{0}, \cdots , d_{31} \}$	32	437 encryption rounds

For the DA1 attack, it is possible to design distinguishers for cubes, whose sizes range from 3 to 20 bits. They work if an adversary is able to observe the keystream after the full initialisation phase (with 2176 rounds). Note that after initialisation, TinyJAMBU employs a set of permutation rounds before producing the keystream. We extend DA1 by including additional permutation rounds (reduced) in the encryption phase of TinyJAMBU. The attack extension is referred to as DA2. For the DA2 attack, we find random distinguishers from a cube space of $2^{96}$, which use 15 and 25 bit cubes. They work for the total number of 2592 rounds. We also show a DA2 that selects cube from a reduced cube space of $2^{32}$. The attack works for up to 2614 rounds with a 18-bit cube.

The DA3–DA5 attacks need 32-bit deterministic cubes. Our experimental results indicate that after 437 rounds, every output bit is affected by the 32-bit cube tester. In other words, all the output keystream bits are expected to depend on the 32-bit cube variables after 437 permutation rounds. Therefore, 437 encryption rounds can be considered as the upper bound for the 32-bit cube tester.

We have also applied two key recovery attacks KRA1 and KRA2. These two attacks are implemented against the initialisation phase of the cipher. For the KRA1, it is possible to recover eight bits of the secret key if an adversary is able to observe the keystream after the full initialisation phase (2176 rounds). The KRA2 identified several linear superpolies for 2592–2604 rounds. Our results show that KRA2 works up to 2604 rounds when the target is recovering at least one bit of the secret key. To the best of our knowledge, our results obtained for TinyJAMBU are the first third-party analysis that produces experimentally verifiable outcomes.

## Cube attack

A cube attack is a relatively recent cryptanalytic technique. To describe it, we follow the presentation given by Dinur and Shamir at EUROCRYPT 2009^8^. The idea behind the attack is to represent a keystream output by a polynomial over secret and public variables. In the cube attack, we assume that an adversary can evaluate the polynomial for public variables. The evaluation allows the adversary to reduce the degree of the polynomial. For AEAD stream ciphers, public variables include bits of the initialisation vector, associated data, and plaintext. It is assumed that the public variables can be chosen by the adversary in an arbitrary way. Unlike algebraic attacks, cube attacks treat the keystream polynomial as a black box.

Suppose that an adversary is able to access a keystream polynomial of a cipher. The polynomial is defined over the binary field *GF*(2). It depends on both secret-key variables $K=\{k_{0}, \cdots , k_{i-1}\}$ and public variables $V=\{v_{0}, \ldots , v_{j-1}\}$. Consider a keystream polynomial ${\mathcal {P}}$ of a degree *deg* over *i* secret and *j* public variables. Define a maxterm $t_{I}$ of the polynomial ${\mathcal {P}}$ as a term whose all variables are public. The term variables are pointed by a collection of indices $I \subseteq \{1, \cdots , j\}$. The variables indexed by *I* are called a cube ${\mathcal {C}}$. The polynomial ${\mathcal {P}}$ can be written as

1 $$\begin{aligned} {\mathcal {P}}(k_{0}, \cdots , k_{i-1}, v_{0}, \cdots , v_{j-1}) \equiv t_{I}.{\mathcal {P}}_{S(I)} + q(k_{0}, \cdots , k_{i-1}, v_{0}, \cdots , v_{j-1}), \end{aligned}$$

where each term of $q(k_{0}, \cdots , k_{i-1}, v_{0}, \cdots , v_{j-1})$ does not contain at least one public variable from the maxterm $t_{I}$. ${\mathcal {P}}_{S(I)}$ is called a superpoly of the index set *I* if it does not contain any constant or any term that has a common factor with the maxterm $t_{I}$. We denote the cardinality of *I* by |*I*| and the size of a cube by $\ell _{c}$. Observe that $|I|=\ell _{c}$. Interestingly enough, if $|I|=deg-1$, then the degree of the superpoly ${\mathcal {P}}_{S(I)}$ is guaranteed to be linear.

Cube attacks work by summing the values of a polynomial ${\mathcal {P}}$ over all possible $2^{|I|}$ Boolean values for variables indexed by *I* (or alternatively over all values of the cube). If the cube is big enough, i.e., $\ell _c=deg-1$, then the degree of ${\mathcal {P}}$ is reduced to one. This means that the superpoly ${\mathcal {P}}_{S(I)}$ becomes linear. If we repeat the above procedure many times but for different cubes, we can generate a system of linear equations involving the secret variables. After a sufficient number of equations, we can solve a system of linear equations and discover the secret variables/key. In general, the cube attack is run in two stages, namely pre-processing and online.

### Pre-processing stage

This stage is executed under an assumption that a description of a stream cipher is public. Consequently, our adversary has access to both public and secret variables and can manipulate them. Our goal is to identify cubes that generate linear superpolies for secret key variables. Since a keystream polynomial ${\mathcal {P}}(K, V)$ form is not known, it is necessary to estimate the degree of ${\mathcal {P}}(K, V)$. This should give us some idea about cube sizes for which we can expect a linear superpoly. We can start from random cubes of small sizes. To choose a random cube ${\mathcal {C}}$ of size $\ell _c$, we select a collection of indices $I_c \subseteq \{0, \cdots , vlen-1\}$ at random, where *vlen* denotes the length of the initialisation vector *V* and $\ell _c=|I_c|$. Consider a keystream polynomial ${\mathcal {P}}_{{\mathcal {C}}}(K, V)=\sum _{{\mathcal {C}}} {\mathcal {P}}(K, V)$ that results from summing ${\mathcal {P}}(K, V)$ over all values of the cube ${\mathcal {C}}$. It is expected that if we have chosen a “right” cube, then ${\mathcal {P}}_{{\mathcal {C}}}(K, V)={\mathcal {P}}_{S(I)}$ is a linear combination of secret variables $\{k_{0}, \cdots , k_{klen-1}\}$, where *klen* is the length of the secret key *K*. To identify the right cube, we need a linearity test for ${\mathcal {P}}_{{\mathcal {C}}}(K, V)$.

We use the BLR test^19^ to check if a polynomial ${\mathcal {P}}_{{\mathcal {C}}}(K, V)$ is linear. The test verifies whether the following relation holds:

2 $$\begin{aligned} {\mathcal {P}}_{{\mathcal {C}}}(K_{0}, V) + {\mathcal {P}}_{{\mathcal {C}}}(K_{1}, V) + {\mathcal {P}}_{{\mathcal {C}}}(K_{2}, V) = {\mathcal {P}}_{{\mathcal {C}}}(K_{1}+K_{2}, V), \end{aligned}$$

where $K_{0} = {\{0\}}^{klen}$ and $K_{1}$, $K_{2}$ are fixed and random bits. If the BLR test is run *n* times, then we can conclude that ${\mathcal {P}}_{{\mathcal {C}}}(K, V)$ is linear with probability $1-2^{-n}$. By choosing a big enough *n* (say $n=100$), we can guarantee the polynomial is linear (with probability $1-2^{-100}$).

Once we get a linear ${\mathcal {P}}_{{\mathcal {C}}}(K, V)={\mathcal {P}}_{S(I)}$, it can be written in its algebraic normal form (ANF) as follows

3 $$\begin{aligned} {\mathcal {P}}_{S(I)}(K) = \alpha _{-1} + \alpha _{0}k_{0} + \alpha _{1}k_{1} + \cdots + \alpha _{klen-1}k_{klen-1}, \end{aligned}$$

where public variables from $V\setminus {\mathcal {C}}$ are set to zero. We know the above representation but we do not know the binary coefficients $\alpha _i$; $i=-1,0,\ldots ,klen-1$. We can determine the coefficients by running $klen+1$ cube experiments

$$\begin{aligned} {\mathcal {P}}_{S(I)}(K=(0,\ldots ,0)) &= {} \alpha _{-1}\\ {\mathcal {P}}_{S(I)}(K=(0,\ldots , \underbrace{1}_{\text{ i-th }},0,\ldots ,0) )&= {} \alpha _{-1} +\alpha _i \text{ for } i=0,\ldots , klen-1 \end{aligned}$$

There is an interesting case when ${\mathcal {P}}_{S(I)}$ stays constant (0 or 1) for all secret keys. Then the polynomial ${\mathcal {P}}_{S(I)}$ is called a distinguisher that allows to differentiate the cipher from a truly random one. Cubes that generate distinguishers are called cube testers^9^.

### Online stage

To execute this stage, it is assumed that an adversary has access to an implementation of the cipher in hand. It can manipulate public variables but cannot see secret ones. Furthermore, we suppose that it has successfully executed the pre-processing stage. In other words, the adversary has discovered $klen+1$ linearly independent superpolies ${\mathcal {P}}_{S(I_j)}$, where each ${\mathcal {P}}_{S(I_j)}$ corresponds to its cube ${\mathcal {C}}_j$. Thus, it can write the following system of equations:

4 $$\begin{aligned} {\mathcal {P}}_{S(I_j)}(K) = \alpha _{-1,j} + \alpha _{0,j}k_{0} + \alpha _{1,j}k_{1} + \cdots + \alpha _{klen-1,j}k_{klen-1}, \end{aligned}$$

where $j=1,\ldots , klen+1$. The values on the left hand side are calculated for the corresponding cubes. As the coefficients $\alpha _{i,j}$ have been determined at the pre-processing stage, the adversary can solve the system from Equation (4) using Gaussian elimination, for example. This concludes the cube attack as the adversary has been able to calculate the secret key *K*.

## Overview of TinyJAMBU

TinyJAMBU^4^ is a family of AEAD sponge-based stream ciphers. The family includes three members: TinyJAMBU-128, TinyJAMBU-192 and TinyJAMBU-256. As we investigate the resistance of TinyJAMBU-128 against cube attacks, our description is focused on TinyJAMBU-128 only.

### Specification of TinyJAMBU-128

TinyJAMBU-128 uses a 128-bit key $K = \{k_{0}, \cdots , k_{127}\}$ and a 96-bit nonce $V= \{v_{0}, \cdots , v_{95}\}$. In the heart of the cipher, there is a 128-bit nonlinear feedback shift register (NFSR). An internal state of NFSR at clock *t* is denoted by $B_t=\{b_{0}^t,b_{1}^t, \cdots ,b_{127}^t\}$. The NFSR state is updated by a nonlinear combination of register bits and a cryptographic key. Unless specified otherwise, a block refers to a group of 32 bits. In particular, the third 32-bit block $\{b_{64},b_{65},\cdots ,b_{95}\}$ of the NFSR is referred to as a keystream. The block is XOR-ed with a plaintext block and they produce the respective ciphertext block. The last 32-bit block $\{b_{96},b_{97},\cdots ,b_{127}\}$ of the NFSR absorbs via XOR all the cipher inputs, i.e. a nonce, associated data and plaintext blocks. The cipher also employs 3-bit constants denoted by *FrameBits* to indicate different phases of cipher operations.

### TinyJAMBU-128 state update function

TinyJAMBU-128 follows a sponge^20^ structure with iterations that use a keyed permutation $P_{r}$. The permutation is implemented using NFSR, whose state update function is described by Algorithm 1. The function takes the five state bits ($b_0$, $b_{47}$, $b_{70}$, $b_{85}$, $b_{91}$) and a bit of the key *K* and produces a feedback bit that becomes $b_{127}$. The permutation $P_r$ calls Algorithm 1 *r* times. 
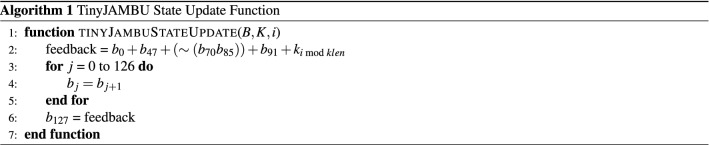


### Operation phases of TinyJAMBU-128

In order to encrypt plaintext blocks, TinyJAMBU-128 goes through four phases, namely, initialisation, associated data processing, encryption and finalisation. For decryption of ciphertext blocks, the cipher proceeds through the same initialisation and associated data processing phases. The next phases are decryption and tag verification, which match the encryption and finalisation phases. As the work describes cube attacks against the first three phases, we briefly discuss them.

#### Initialisation

Algorithm 2 shows a pseudocode of the initialisation phase. It consists of two parts, namely, key and nonce setups. At the key setup, a cryptographic key *K* is loaded into the NFSR by executing $P_{1024}$. During the nonce setup, a nonce is absorbed into NFSR as a 32-bit block. *FrameBits* are set to “1”. For each nonce setup call, the NFSR state is updated by running $P_{384}$ before the nonce blocks are XOR-ed into the NFSR state. Note that the second version of TinyJAMBU-128 employs $P_{640}$ instead of $P_{384}$ during the nonce setup. 
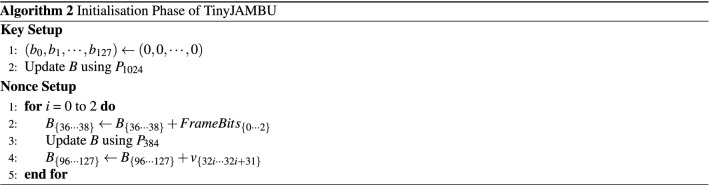


#### Associated data processing

After the NFSR state is initialised, the associated data $AD = AD_{\{0 \cdots adlen-1\}} = \{d_{0}, \cdots , d_{adlen-1}\}$ are processed block by block, where *adlen* is the length (number of bits) of the associated data. Algorithm 3 details steps of the associated data processing. The NFSR state is first updated by running the permutation $P_{384}$, which is followed by loading the 32-bit associated data into $B_{\{96 \cdots 127\}}$. Note that if the length *adlen* of associated data is not a multiple of 32, then additional steps are required to process the last partial block of associated data (refer to the original description of TinyJAMBU for details). *FrameBits* in this phase are set to “3”. Similarly to the nonce setup, the second version of TinyJAMBU-128 applies $P_{640}$ instead of $P_{384}$ for the associated data processing. 
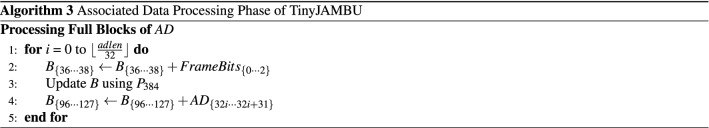


#### Encryption

Algorithm 4 illustrates the encryption phase. Encryption directly follows the associated data processing phase. *FrameBits* are set to “5” during the encryption. Plaintext bits are processed block by block. Let $M = \{m_{0}, \cdots , m_{mlen-1}\}$ denote the plaintext of length *mlen*. Given a plaintext block $M_{\{32i \cdots 32i+31 \}}$, then it is encrypted by XOR-ing it with $B_{\{64 \cdots 95\}}$, which is a keystream block extracted from the NFSR state. Note that two consecutive plaintext block encryptions are separated by the NFSR state update. The update is done by calling $P_{1024}$. If the length of plaintext *mlen* is not a multiple of 32, then the remaining bits of plaintext require further processing (refer to the original description of TinyJAMBU for details). 
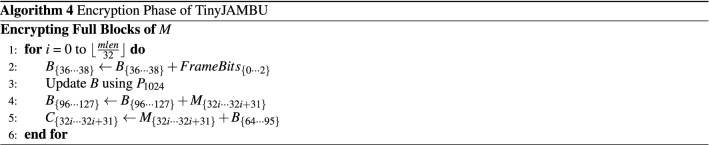


## Cube attack against TinyJAMBU

Observe that nonce, associated data and plaintext bits are used to constantly update the NFSR state. Clearly, the authors have intended to increase dependencies among all bits involved in the initialisation, associated data processing and encryption phases. Besides, the cryptographic key *K* is always used for each state update. Consequently, each output bit of the permutation $P_r$ can be seen as a complex function of all input bits. They include bits of the NFSR state, the key, the nonce, the associated data and the plaintext.

As a significant part of the bits are public, there are many options for selecting cubes at the pre-processing stage. We implement five distinguishing attacks DA1–DA5 and two key recovery attacks KRA1–KRA2. They cover the three cipher phases: initialisation, the associated data processing and encryption. We need to pay attention to the third block $B_{\{64 \cdots 95\}}$ of the NFSR state as it plays the role of keystream. We aim to identify bits of a keystream block that, when used in a cube, produce either linear superpolies or constants.

Algorithm 5 details steps in the pre-processing phase of our generic cube attack against the cipher. Its goal is to identify cube testers or cubes with linear superpolies. As we do not have any information about appropriate cube sizes, we test different cube sizes $\ell _{c}$. For each cube size, the resulting superpolies of random cubes are tested for linearity. In Algorithm 5, the C++ built-in function *rand*() is used to generate the arbitrary selection of cubes. Note that the pre-processing stage is needed to perform only once. The pre-processing stage is already completed for the cubes identified for TinyJAMBU in this paper. Hence, an adversary does not require to compute it again and can proceed directly to the online stage without performing this stage. Algorithm 5 also shows the pseudocode for steps performed at the online stage. The online stage is needed to perform every time the cipher is re-keyed (for a key recovery attack). The adversary needs to use a known plaintext attack or a chosen plaintext attack model depending on the operation phases of TinyJAMBU to which the attack is applied. These are common assumptions within security models for performing the analysis of ciphers including the cube attack. Our implementations of cube attacks are applied to round-reduced variants of TinyJAMBU. All results have been experimentally verified (see Ref.^21^ for the source codes and detailed experimental results from all our implementations).

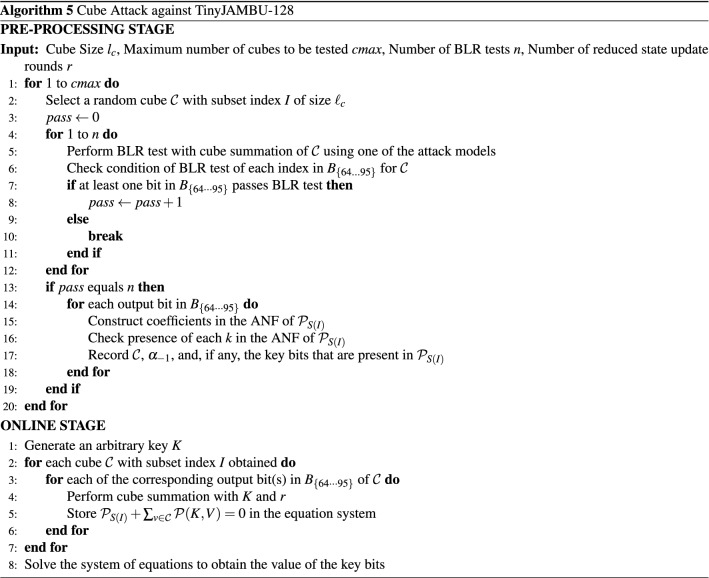



### Description of attack process in the initialisation phase

Out of five distinguishing attacks investigated and implemented in the work, our two attacks, DA1 and DA2 are in the initialisation phase of TinyJAMBU-128. The key recovery attacks KRA1 and KRA2 are also against the initialisation phase of TinyJAMBU-128. The details of the attacks are shown in Table 2. We assume that they are applied at clock $t=0$ and cubes are chosen from the nonce bits only. As a 32-bit keystream block depends on key and nonce bits, we intend to find cubes (defined over nonce bits only) whose superpolies are linear and depend on some key bits.

**Table 2:** Assumptions for DA1, DA2, KRA1, KRA2.

DA1 and KRA1: reduced-round initialisation phase	DA2 and KRA2: reduced-round initialisation phase with additional encryption rounds
Starting state of the attack: $B_0$	Starting state of the attack: $B_0$
Cubes randomly chosen from first 64 bits of nonce: $\{v_0,\cdots ,v_{63}\}$	Cubes chosen randomly from all 96 bits of nonce : $\{v_0, \cdots ,v_{95}\}$, or chosen randomly from the last block of nonce : $\{v_{64}, \cdots ,v_{95}\}$
Steps taken: 1. Cube is XOR-ed into the state. 2. *B* goes through reduced permutation: $\bullet$ key setup, $P_{r_{1} = 1024}$ $\bullet$ nonce setup, $P_{r_{2} = 384}$ 3. Keystream is observed after $P_{r_{2}}$.	Steps taken: 1. Cube is XOR-ed into the state. 2. *B* goes through reduced permutation: $\bullet$ key setup,$P_{r_{1} = 1024}$ $\bullet$ nonce setup,$P_{r_{2} = 384}$ $\bullet$ additional encryption rounds, $P_{r_{3}}$ 3. Keystream is observed after $P_{r_{3}}$.

Consider DA1 and KRA1 from Table 2. We assume that the cipher goes through initialisation but skips the associated data processing and encryption phases. In other words, we can observe the keystream immediately after the permutation round of the initialisation phase. We choose cubes at random from a 64-bit nonce. Note that due to our assumptions, the NFSR state does not go through any permutation rounds after the last 32 bits of the nonce is XOR-ed into the last block of the state. This means that the last 32 bits of the nonce do not get mixed into the keystream block. Consequently, the keystream does not contain any variables from the last 32 bits of the nonce. Trivially, if we include variables from the last 32 bits of the nonce, then we get a distinguisher as cube summation must give us a constant.

Note that according to the specification of TinyJAMBU-128, the cipher goes through 1024 rounds of permutation before keystream bits can be observed. Thus, DA1 and KRA1 are extended to DA2 and KRA2, respectively (see Table 2). These attacks are against a cipher that includes $r_3$ additional permutation rounds (reduced) at the encryption phase. This means that the cipher does not absorb any associated data, i.e., processing of associated data is skipped. This also implies that keystream bits depend on both the key and nonce bits. So cubes can be selected from all 96 bits of the nonce. For DA2 and KRA2, the cipher uses the full initialisation phase, i.e, $r_{1} = 1024$ and $r_{2} = 384$. However, the number $r_{3}$ of encryption permutation rounds is reduced.

The DA1 and KRA1 are performed to identify the dependency of the output function with the first 64 nonce bits immediately after the initialisation phase. These two attacks determine whether an adversary can perform an attack by selecting cubes from the first 64 nonce bits if the keystream was to be observed immediately after the initialisation. Hence, it is assumed that the keystream can be observed without going through the encryption phase for these two attacks. In contrast, the DA2 and KRA2 are performed to identify the dependency of the output function with the nonce bits, including the last block of the nonce. For these two attacks, after completing the initialisation phase, reduced encryption permutation rounds are assumed to be performed since the cubes may now contain the last block of the nonce. The last block of the nonce will result in trivial distinguishers if these additional encryption permutation rounds are not performed. Note that the associated data processing phase is an optional phase for TinyJAMBU; that is, if there is no associated data, then this phase will be skipped. The associated data processing phase is assumed to be skipped for all these four attacks as no keystream is output during this phase.

#### Experimental results for DA1 and KRA1

We have implemented DA1 and KRA 1 as described by Algorithm 5. For a given cube size, we choose $cmax = 5000$ random cubes. For each cube, we run 50 BLR linearity tests. We have found many cube testers, whose sizes range from $\ell _{c} =3$ to $\ell _{c} = 20$. The total number of permutation rounds employed in the cipher is $1024+384\times 3=2176$. A sample of cube testers found is presented in Table 3. We only list cubes up to size 12 in this table. The table details: cube size (the first column), a collection of cube indices (the second column), a collection of keystream bits corresponding to the cube tester (the third column) and the number of superpolies for the given cube (the fourth column). Some additional statistics are presented in Table 4. Note that the complexity of the DA1 very much depends on the size of a cube. This is to say that it ranges from $\Theta (2^3)$ to $\Theta (2^{20})$.

**Table 3:** Examples of cube testers found using DA1.

Cube size, ${l_c}$	Cube (nonce) indices, *I*	Keystream indices	No. of affected indices
3	51, 52, 63	70	1
4	37, 52, 57, 62	64	1
5	18, 48, 55, 60, 61	67, 73	2
6	24, 39, 52, 53, 58, 63	65, 70	2
7	32, 33, 41, 48, 52, 54, 59	66, 72	2
8	1, 14, 40, 47, 53, 54, 59, 60	66, 71, 72	3
9	0, 27, 30, 46, 52, 58, 60, 61, 63	64, 65, 70	3
10	19, 32, 45, 47, 49, 50, 56, 57, 61, 63	64, 66, 68, 75	4
11	10, 11, 21, 22, 41, 50, 55, 56, 57, 62, 63	69, 74, 75, 81	4
12	13, 16, 17, 45, 48, 49, 53, 56, 57, 60, 61, 62	64, 65, 67, 68, 74	5

**Table 4:** Summary of the results of cube testers found using DA1.

Cube size ${l_c}$	Maximum count of output indices	Number of cube testers	Percentage (% per 5000 cubes)
4	1	16	0.32
5	2	56	1.12
6	2	121	2.42
7	2	155	3.1
8	3	289	5.78
9	3	409	8.18
10	4	606	12.12
11	4	793	15.86
12	5	1092	21.84
13	7	1343	26.86
14	9	1587	31.74
15	10	2064	41.28
16	11	2325	46.5
17	12	2739	54.78
18	16	3106	62.12
19	15	3471	69.42
20	18	3753	75.06

We also found a small set of cubes that resulted in non-constant superpolies. These superpolies are used to implement the KRA1. These are listed in Table 5. The cubes for KRA1 range from $l_c = 3$ to $l_c = 13$ and can be used to recover eight bits of the secret key after 2176 rounds of the initialisation phase. The complexity of solving these equations is negligible.

**Table 5:** Examples of superpolies found using KRA1.

Cube (nonce) indices, *I*	Keystream indices	Superpoly
40, 53, 54	66	$k_{15}+k_{35}+k_{37}+k_{77}+k_{84}+k_{94}$
39, 52, 53, 58	65	$k_{45}+k_{77}$
32, 47, 51, 52	64	$k_{9}+k_{20}+k_{34}+k_{35}+k_{61}+k_{76}+k_{106}$
1, 44, 60, 61	73	$k_{4}+k_{8}+k_{13}+k_{33}+k_{60}+k_{69}+k_{104}+k_{121}$
32, 51, 52, 58, 59, 60	71	$k_{30}$
40, 47, 52, 60, 62, 63	65	$k_{85}+k_{125}$
13, 20, 22, 23, 33, 34, 40, 44, 45, 55, 56, 60	73	$k_{8}+k_{99}$
11, 15, 17, 20, 28, 31, 35, 40, 41, 55, 58, 61, 62	74	$k_{3}+k_{38}+k_{110}$

The experiments demonstrate that the initialisation phase of the cipher provides a relatively low diffusion. This is due to the fact that the cipher iterates 384 times the permutation *P* after loading the second block of the nonce. This number is definitely too low. Note that the authors of the cipher have now increased this number to 640, which improves diffusion during the initialisation phase.

#### Experimental results for DA2 and KRA2

We have also conducted experiments for the DA2 and KRA2 attacks. In this case, we assume that the cipher includes the full initialisation phase together with a reduced number of permutation rounds $P_{r_{3}}$ at the encryption phase. Note that after initialisation, the NFSR state goes through the permutation $P_{r_{3}}$. It means that the last 32 bits of the nonce bits get mixed with other bits before keystream bits become observable. This implies that in the attack, we can choose cubes from all 96 bits of the nonce.

The attack follows the steps given by Algorithm 5. For a given cube size, we choose $cmax = 5000$ random cubes. Given a cube, we determine its superpoly and check its linearity by running 50 BLR tests. We begin with $r_3 = 384$ rounds and then, we keep increasing the number $r_3$ by a multiple of 32, i.e. $r_3 = 384, 416, 448, \ldots$. We refer to this as DA2 with random cubes selected over the full cube space. During our experiments, we are able to find many cube testers of size 15 for the permutation $P_{384}$ and one cube tester of size 25 for the permutation $P_{416}$. We have also conducted experiments for the permutation $P_{448}$ with cube sizes up to $\ell _{c} = 40$. However, we have failed to find any.

A sample of cube testers of size 15 and the only cube tester of size 25 are given in Table 6. Cube testers of size 15 are able to distinguish the cipher from a truly random one if the cipher uses no more than 2560 rounds of the permutation *P*. The best result we got for DA2 with random cube selection from the full cube space is the cube tester of size 25 that works for the cipher with 2592 rounds of the permutation *P*.

**Table 6:** Examples of cube testers found using DA2 with full cube space $V_{\{0 \cdots 95 \}}$.

Cube size ${l_c}$	Cube (nonce) indices, *I*	Additional encryption rounds, $r_3$	Indices of the keystream	No. of affected indices
15	15, 21, 22, 32, 43, 68, 71, 72, 81, 85, 88, 90, 93, 94, 95	384	64, 65, 68, 74, 79, 80	6
15	2, 7, 10, 18, 21, 25, 26, 27, 28, 39, 40, 42, 86, 87, 93	384	73	1
15	0, 10, 12, 21, 29, 49, 58, 60, 78, 79, 80, 81, 82, 86, 90	384	65, 66	2
15	3, 8, 21, 28, 36, 37, 53, 60, 63, 65, 72, 82, 84, 88, 93	384	68	1
15	2, 3, 19, 32, 35, 38, 55, 68, 75, 78, 81, 82, 87, 89, 90	384	67	1
25	8, 23, 25, 31, 35, 36, 39, 40, 54, 56, 57, 65, 68, 71, 72, 73, 74, 76, 78, 83, 84, 87, 90, 93, 94	416	69	1

Next, we have tried to find cubes for an arbitrary number $r_3$, not necessarily a multiple of 32. As the last block of the nonce is the last to be XOR-ed into the NFSR state, one can argue that the block bits are not as thoroughly mixed with other bits. So it is reasonable to choose cubes taking as many as possible bits from the last block. This approach should eliminate the maxterms of the corresponding superpoly that are not mixed well with the last block of the nonce. This approach has been verified experimentally. We refer to this as DA2 with reduced cube space. We find that the 32-bit cube $\{ v_{64}, \cdots , v_{95} \}$ works up to $r_{3}=437$ rounds of the permutation *P* and results in a distinguisher. As a result, with this method, we have got cube testers that allow to distinguish the cipher with 2613 rounds of *P* from a truly random cipher.

##### DA2 with smaller cube sizes and extension to a key recovery attack

The 32-bit cube for 437 rounds DA2 is a distinguisher. This means the cube size is too large. We use two techniques for extending the experiments to identify DA2 with smaller cube sizes and possible extensions to a key recovery attack (KRA2). For these experiments, we select the cube bits from a reduced set of nonce bits (last block of the nonce). Other nonce blocks are only included in the cube space when the cube size is larger then 32 bits. In other words,

- for cubes of size $\ell _{c} \le 32$, the cube bits are selected from the last block bits $V_{\{64 \cdots 95\}}$ only; whereas,
- for cubes of size $\ell _{c} > 32$, the last block bits $V_{\{64 \cdots 95\}}$ are always present in the cube. The remaining cube bits are chosen randomly from the bits $V_{\{0 \cdots 63\}}$.

##### Technique 1

We conducted experiments by gradually reducing the size of the 32-bit cube. The degree of a superpoly is expected to increase (roughly by 1) when the size of the corresponding cube is reduced by 1. For each cube size, depending on the cube space, we tested $cmax=5000$ to $cmax=100{,}000$ superpolies generated from random cubes of the given size. The superpolies are tested for at least 200 linearity tests. This process enabled us to find additional distinguishers for DA2 with much smaller cube sizes. We also found a small number of non-constant superpolies with these experiments. Overall, with this process, we found cubes of sizes in between $l_c = 13$ to $l_c =21$. These cubes work for encryption round in between $r_3= 416$ to $r_{3} = 437$.

##### Technique 2

Cubes obtained using Technique 1 above are of relatively smaller sizes ($\le 21$). For any such cube sizes, the search space is relatively small and the search time is fast due to the smaller cube sizes. It is possible to exhaustively test the entire cube space for such cases. We conducted a set of experiments by reducing the cube sizes further and then enumerating through the entire cube spaces of the reduced cube sizes. Algorithm 6 details the steps of this process. Using steps in Algorithm 6, we have obtained additional distinguishers and non-constant superpolies. 
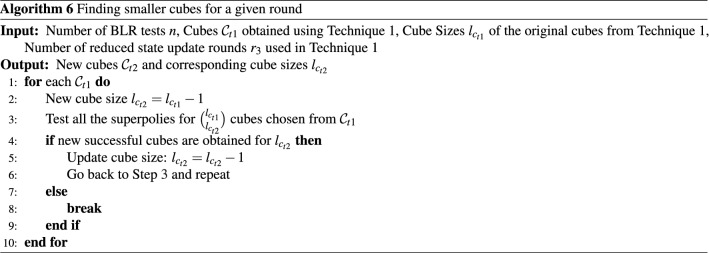
 A sample of the cube testers and non-constant superpolies that are obtained using the above two techniques are listed in Tables 7 and 8, respectively.

**Table 7:** Examples of cube testers for DA2 with reduced cube space $(V_{\{64, \cdots , 95\}})$.

Cube Size, ${l_c}$	Cube (Nonce) Indices, *I*	Enc. Rounds, $r_3$	Keyst. Indices, $z_{i}$
8	69, 70, 71, 76, 81, 86, 91, 92	416	67
9	64, 69, 70, 71, 76, 81, 86, 91, 92	416	67
13	66, 67, 68, 69, 71, 73, 83, 87, 88, 90, 91, 94, 95	416	64
14	67, 68, 69, 72, 73, 74, 80, 81, 84, 85, 88, 90, 91, 94	416	64
15	65, 66, 69, 70, 71, 73, 74, 77, 82, 83, 85, 88, 89, 90, 92	416	65
15	64, 66, 70, 72, 73, 75, 78, 79, 82, 83, 86, 87, 89, 92, 93	420	65
11	65, 72, 78, 79, 83, 84, 86, 90, 92, 93, 95	425	66
15	64, 65, 70, 72, 73, 77, 78, 79, 83, 84, 86, 90, 92, 93, 95	425	66
21	64, 65, 67, 69, 70, 71, 72, 74, 76, 78, 79, 80, 81, 83, 84, 85, 86, 88, 90, 91, 94	430	68
14	64, 65, 70, 72, 76, 77, 78, 81, 85, 86, 87, 88, 92, 95	430	69
18	67, 68, 69, 70, 72, 73, 75, 79, 80, 81, 83, 84, 85, 88, 89, 90, 91, 95	437	65
18	66, 67, 68, 72, 73, 75, 77, 79, 81, 82, 83, 84, 87, 88, 89, 90, 93, 94	437	64
21	64, 67, 68, 69, 71, 73, 74, 75, 76, 79, 80, 81, 83, 84, 88, 89, 90, 91, 92, 94, 95	437	65
21	64, 66, 67, 68, 72, 73, 75, 77, 79, 81, 82, 83, 84, 87, 88, 89, 90, 91, 92, 93, 94	437	64

Recall that the register bits that are used for keystream, i.e., $\{ b_{64}^{t}, \cdots , b_{95}^{t} \}$, are updated by shifting the contents of the register bits $\{ b_{65}^{t-1}, \cdots , b_{96}^{t-1} \}$. Therefore, any successful cube for a keystream bit $b_{i}^{t}$ will also work for the keystream bit $b_{i-1}^{t+1}$. With technique 1, surprisingly we found some DA2 cubes of sizes $l_{c} \le 31$ that are successful for the keystream bit $b_{65}^{437}$ (see Table 7). This means the same cube will also be successful for the keystream bit $b_{64}^{438}$, i.e., will work up to $r_{3} = 438$ rounds. Experimental results confirm this observation. The best distinguisher for DA2 works until $r_{3} = 438$ rounds with a cube size of 18. As a result, with this method, we have obtained a cube tester that allows us to distinguish the cipher with 2614 rounds of *P* from a truly random cipher. We think that the smaller cube size that works for $r_3 = 438$ rounds is due to the structure of the corresponding output polynomial. To illustrate an example where a superpoly may pass for a smaller cube but fails for a larger cube, let us consider a hypothetical output function ${\mathcal {P}}_{h} (K, V) = v_0v_1k_0k_1+v_0v_2k_0k_1$. The cube summation $\sum _{v_{0}}{\mathcal {P}}_{h}$ over the cube $v_0$ will pass the linearity test. However, a larger cube $v_{0}v_{1}$ or $v_{0}v_{2}$ of size 2 will fail the linearity test in this case. To check for such cases for $r_{3} \ge 438$, we further tested cubes with sizes $l_{c} < 32$. However, we did not find any such cubes for $r_{3}$ rounds beyond 438.

For KRA2, we obtained several non-constant superpolies for $r_{3} = 416$ to $r_{3} = 428$ rounds. However, some superpolies are repeated for different cubes, i.e., some equations are the same. The cube sizes for these superpolies ranges between 9 to 16. Notice for Table 8 that most of these superpolies contains only a single variable. Therefore, during the online phase, the cube summation results itself will output the values of most of these superpolies. Overall, the best cube for KRA2 works up to $r_3 = 428$ rounds when the target is at least a single bit recovery of the key.

**Table 8:** A list of superpolies found using KRA2 with reduced cube space $V_{\{64, \cdots , 95\}}$.

Cube size, ${l_c}$	Cube (Nonce) Indices, *I*	Rounds, $r_3$	Keyst. Index $z_{i}$	Superpoly ${\mathcal {P}}_{S(I)}$
9	64, 70, 71, 76, 77, 82, 87, 92, 93	416	68	$k_{15}$
9	69, 70, 75, 76, 81, 86, 91, 92, 94	416	67	$k_{90}$
9	65, 71, 72, 77, 85, 86, 92, 93, 95	416	68	$k_{15}$
10	69, 70, 74, 75, 81, 84, 86, 90, 91, 93	416	66	$k_{90}$
10	64, 70, 71, 72, 77, 86, 87, 92, 93, 95	416	68	$k_{15}$
10	65, 72, 76, 77, 78, 85, 86, 88, 92, 93	416	68	$k_{15}$
10	65, 72, 76, 77, 80, 85, 86, 88, 92, 93	416	68	$k_{15}$
10	71, 72, 77, 78, 82, 87, 88, 89, 93, 94	416	69	$k_{105}$
11	64, 70, 71, 73, 75, 76, 82, 84, 85, 91, 92	416	67	$k_{3}$
11	66, 67, 69, 72, 73, 80, 82, 83, 84, 88, 89	416	64	$k_{78}$
11	66, 73, 74, 78, 81, 83, 86, 87, 88, 93, 94	416	69	$k_{31}$
11	69, 74, 75, 79, 80, 81, 88, 89, 91, 92, 95	416	71	$k_{36}$
12	65, 72, 73, 74, 79, 80, 84, 87, 88, 89, 92, 94	416	70	$k_{101}$
12	69, 71, 74, 75, 79, 80, 81, 88, 89, 91, 92, 95	416	71	$k_{36}$
12	69, 74, 75, 79, 80, 81, 88, 89, 91, 92, 93, 95	416	71	$k_{36}$
12	73, 74, 76, 79, 80, 81, 84, 88, 89, 90, 91, 95	423	64	$k_{43}$
13	69, 71, 74, 75, 79, 80, 81, 88, 89, 91, 92, 93, 95	416	71	$k_{36}$
14	64, 65, 70, 71, 72, 75, 77, 79, 81, 85, 86, 91, 92, 93	416	68	$k_{15}$
14	68, 71, 72, 73, 74, 79, 82, 83, 84, 85, 89, 90, 92, 94	416	65	$k_{35}$
14	65, 66, 67, 72, 73, 74, 78, 85, 86, 87, 89, 92, 93, 94	416	69	$k_{8} + k_{28}$
14	66, 67, 69, 72, 73, 79, 80, 82, 83, 84, 86, 88, 89, 92	416	64	$k_{57} + k_{59} + k_{89}$
15	65, 72, 73, 74, 77, 82, 83, 84, 85, 86, 87, 88, 93, 94, 95	416	69	$k_{112}$
15	72, 74, 77, 78, 79, 80, 81, 84, 85, 86, 87, 88, 90, 94, 95	428	64	$k_{0}$
15	71, 72, 74, 78, 79, 80, 81, 84, 85, 86, 88, 90, 91, 94, 95	428	64	$k_{26}$
16	71, 72, 74, 75, 78, 79, 80, 81, 84, 85, 86, 88, 90, 91, 94, 95	428	64	$k_{26}$
16	72, 74, 75, 78, 79, 80, 81, 83, 84, 85, 86, 88, 90, 91, 94, 95	428	64	$k_{39}$
16	65, 66, 72, 74, 76, 78, 79, 80, 81, 83, 84, 85, 88, 90, 94, 95	428	64	$k_{11}$

#### Overall comments on the results of DA2 and KRA2

It is worth noticing that the original TinyJAMBU-128 takes 3200 rounds of the permutation *P*. We count the number of rounds executed during initialisation and encryption of the first plaintext block. It appears that the cipher (its first version) leaves a relatively small security margin, which is $3200-2614=586$ rounds.

The computational complexity of the DA2 attack varies from $\Theta (2^{8})$ to $\Theta (2^{32})$. Compared to the complexity of DA1, the computation overhead for DA2 is significantly higher. This difference is the result of a bigger number of rounds in the attacked cipher that includes the initialisation and encryption phases. Our experiments confirm the necessity to separate processing of two consecutive 32-bit input blocks by a sufficiently big number of rounds of *P*. The increment of the number $r_2$ of *P* rounds from 384 (for TinyJAMBUv1) to 640 (for TinyJAMBUv2) strengthens the cipher as it increases both diffusion of bits and algebraic degree of keystream functions. The margin for DA2 with random cubes from full cube space $(2^{96})$ against TinyJAMBUv2 is expected to be higher than the first version of the cipher. For TinyJAMBUv2, the security margin against DA2 with the reduced cube space is expected to be the same as the first version ($3968-3382 = 586$ rounds). The security margin against KRA2 (at least a single bit key recovery) for TinyJAMBUv1 is 596 rounds. The same margin for KRA2 is expected against TinyJAMBUv2.

### Description of attack process in encryption phase

The remaining three attacks DA3–DA5 are applied against a round-reduced cipher. Table 9 specifies the assumptions about round-reduced versions of the cipher. As the key bits are absorbed into the NFSR state during each permutation round, a goal of our attacks is not only to find cube testers but also to recover some bits of the key. As an independent research challenge, we aim to verify the designer’s claim asserting that all bits of the keystream depend on all input bits after 598 rounds of the permutation *P*^4^. For the second version of the cipher (TinyJAMBUv2), the claim has been updated and it says that the full dependence is achieved after 512 rounds^5^.

**Table 9:** Assumptions for DA3, DA4 and DA5.

DA3: Reduced Rounds Encryption Phase Using Plaintext Bits	DA4: Reduced Rounds Encryption Phase Using Nonce Bits	DA5: Reduced Rounds Encryption Phase Using Associated Data Bits
Assumptions: $\bullet$ No associated data $\bullet$ Starting state: $B_{3200}$ $\bullet$ Cube, ${{\mathcal {C}}} \in \{m_{0}, \cdots , m_{31}\}$	Assumptions: $\bullet$ No associated data $\bullet$ Starting state: $B_{2176}$ $\bullet$ Cube, ${{\mathcal {C}}} \in \{v_{64}, \cdots , v_{95}\}$	Assumptions: $\bullet$ Includes associated data $\bullet$ Starting state: $B_{2560}$ $\bullet$ Cube, ${{\mathcal {C}}} \in \{d_{0}, \cdots , d_{31}\}$
Steps taken:		
1. Cube, ${\mathcal {C}}$ is XOR-ed into last 32-bits of state *B*, i.e., $B_{\{96 \cdots 127\}}$.		
2. State *B* goes through reduced permutation rounds $P_{r_3}$ in the encryption phase.		
3. Keystream, $B_{\{64 \cdots B_{95} \}}$, is observed and cube summation is computed after $P_{r_3}$.		

For the DA3 attack, we assume that the cipher runs through the full initialisation phase and the permutation $P_{1024}$ when processing the first 32-bit plaintext block. Note that the associated data processing phase is skipped. Thus the attack starting state becomes $B_{3200}$. The length *mlen* of plaintext is set to 64 bits. A cube is chosen to include the first 32 bits of the plaintext, i.e., $\{m_{0}, \cdots , m_{31}\}$ and the remaining 32 bits of the plaintext are set to zero.

For the DA4 attack, the cipher executes the initialisation phase, where the NFSR state goes through the full $1024+384 \times 3=2176$ permutation rounds. It means that the attack starting state is $B_{2176}$. Note that the associated data processing phase is again skipped. Table 9 shows details of the attack. In particular, cubes are chosen from the last 32 bits $\{v_{64}, \cdots , v_{95}\}$ of the nonce *V*. In the encryption phase, the *FrameBits* are XOR-ed into the state and the state is updated by running $P_{r_{3}}$.

The DA5 attack is similar to DA4. We assume that the cipher executes the initialisation phase (with 2176 permutation rounds) and processes the first 32 bits of associated data (with 384 permutation rounds). Thus, the attack starting state becomes $B_{2560}$. Similarly to DA4, in the encryption phase, *FrameBits* are XOR-ed and the state is updated by the permutation $P_{r_{3}}$ with a reduced number $r_3$. Cubes are selected from the first block of associated data, i.e., $\{d_{0}, \cdots , d_{31}\}$. Table 9 compares our three attacks. The main difference among them is the selection of cubes.

The DA3 and DA4 are similar to the case of DA2, except that in these two cases, the cubes are chosen from the first block of the plaintext or the last block of the nonce, respectively. Hence, similar to DA2, these two attacks also assume to skip the associated data processing phase. However, we note that for DA3, the attack can be performed even if the associated data processing phase is included. The DA3 selects the cube from the plaintext, which occurs after the associated data processing phase. Hence, the additional rounds of the associated data processing phase will not impact the plaintext cubes. The DA5 is performed to identify the dependency of the output function with the associated data bits; hence, the associated data processing phase is included for this attack.

#### Experimental results for DA3, DA4 and DA5

We have implemented the three attacks. Cubes are chosen according to the attack specification (see Table 9). Given a cube, we check the resulting superpoly for linearity using 50 BLR tests. At the same time, the number of permutation rounds of $P_{r_{3}}$ is gradually increased. Consider DA3. We have found a few single bits of the keystream outputs that produce constant superpolies for $r_3=416, 417, 437$. Similar results are obtained for DA4. For the DA5 attack, we get linear superpolies for $r_3=416, 437$. Table 10 summarises our experiments with the three attacks. Note that we did not test all the values for $r_{3}$ between 416 to 437. However, we are confident that cube testers exist for any $r_3$ in the interval (416, 437).

**Table 10:** Experimental results of 32-bit cube for DA3, DA4 and DA5.

Attack	Reduced round, $r_{3}$	Output Indices	Total indices
DA3	416	64, 65, 66, 67, 68, 69, 70,71, 72, 73, 74, 75, 76, 77, 78, 79, 80, 81, 82, 83, 84, 85	22
	417	64, 65, 66, 67, 68, 69, 70, 71, 72,73, 74, 75,76, 77, 78, 79, 80, 81, 82, 83, 84	21
	437	64	1
DA4	416	64, 65, 66, 67, 68, 69, 70, 71, 72, 73, 74, 75, 76, 77, 78, 79, 80, 81, 82, 83, 84, 85	22
	417	64, 65, 66, 67, 68, 69, 70, 71, 72, 73, 74, 75, 76, 77, 78, 79, 80, 81, 82, 83, 84	21
	418	64, 65, 66, 67, 68, 69, 70, 71, 72, 73, 74, 75, 76, 77, 78, 79, 80, 81, 82, 83	20
	437	64	1
DA5	416	64, 65, 66, 67, 68, 69, 70, 71, 72, 73, 74, 75, 76, 77, 78, 79, 80, 81, 82, 83, 84, 85	22
	437	64	1

For all attacks, the largest number of rounds in the encryption phase is $r_{3} =437$. We have also tried bigger values (i.e. $r_{3}\ge 438$). Unfortunately, we could not find any cube and the matching superpoly that passes the BLR test. Note that the 32-bit cube testers allow to tell apart the cipher from a random one only. Although the attacks do not allow to recover any of the key bits, they give an insight into cipher security.

As all cube testers, in the three attacks, require 32-bit cubes, the complexity of the attacks is $\Theta (2^{32})$. Note that the attacks apply a similar approach. It should not be a surprise that the results are also similar. From the result given in Table 10 we see that our cube testers work up to 437 rounds in the encryption phase. This leads us to a conclusion that the cipher has a better security margin than the one claimed by the designers.

The cubes for DA2 and KRA2 can also be applied against DA3 to DA5 by using same or corresponding indices for plaintext in DA3, nonce in DA4 and associated data in DA5. Experimental results verify this observation. So, it is also possible to find cube sizes of 18 (compute corresponding indices from Table 7) for DA3 to DA5 that works for 438 rounds of encryption phase.

## Conclusion

We have investigated the resistance of the TinyJAMBU cipher against cube attacks. The cipher is a finalist of the NIST LWC Project. We have applied five variants of the distinguishing attack: DA1–DA5, and two variants of the key recovery attack: KRA1–KRA2. They all target the first version of the cipher called TinyJAMBU-128. The changes in the second version of the cipher only increase the number of rounds during the nonce-setup, associated data processing, and finalisation; no other changes are made in this version. The first two attacks DA1 and KRA1 are launched against the initialisation phase (that includes 2176 rounds) of the cipher. For DA1, we have been able to find cube testers (distinguishers) with cube sizes ranging from 3 to 20. For KRA1, we have identified non-constant superpolies that can be used to recover eight bits of the secret key. The attack DA2 is an extension of DA1. It is applied against a cipher variant that includes the initialisation phase and 438 encryption rounds. We have found 18-bit cube testers. The KRA2 is applied against a cipher variant that includes the initialisation phase and 428 encryption rounds. Note that the results of DA1 and some results in DA2 (for random cubes from full cube space) are only applicable to TinyJAMBUv1. However, the results from the DA2 with reduced cube space and KRA2 are applicable to both TinyJAMBUv1 and TinyJAMBUv2.

The other three attacks (DA3–DA5) are against cipher variants with the encryption phase. Bits of cubes are chosen from either plaintext, nonce or associated data. We note that for DA3 to DA5, there are some smaller cubes of sizes less than 32 that work up to 438 rounds; however, the superpoly of the 32-bit cube tester do not pass beyond 437 rounds. As a result, we have identified 437 rounds as the upper bound on the number of rounds, for which the attacks work and allow to find 32-bit cube testers. Note that the designers of TinyJAMBUv2 claim that after 512 rounds, all output bits in keystream are affected by all input bits. Based on our results, we expect that the full dependency is achieved after 437 rounds. The conclusion on the full dependency achievement of a 32-bit cube tester after 437 rounds are based on the fact that the deterministic 32-bit cube tester does not pass beyond 437 rounds. That means all these 32 cube variables are present in the output equation obtained after 437 rounds. As the degree of the output function increases significantly after each round, it is expected that the 32-bit cube variables will be present in all the output equations obtained after 437 rounds. Hence, the cube is unlikely to pass beyond 437 rounds.

We emphasize that the results reported in this paper do not threaten the security of TinyJAMBU. We hope that the cubes identified in the work contribute to a better understanding of security strengths and limitations of the cipher.

## Data Availability

The source codes and detailed experimental results for all our implementations can be accessed from: https://github.com/cst1709690/tinyJambuCubeAttack.
